# Current state of prostate cancer treatment in Jamaica

**DOI:** 10.3332/ecancer.2014.456

**Published:** 2014-08-28

**Authors:** Belinda F Morrison, William D Aiken, Richard Mayhew

**Affiliations:** Department of Surgery, University of the West Indies, Kingston, Jamaica

**Keywords:** prostate cancer, Jamaica, outcomes, radical prostatectomy, radiation

## Abstract

Prostate cancer is the commonest cancer in Jamaica as well as the leading cause of cancer-related deaths. One report suggested that Jamaica has the highest incidence rate of prostate cancer in the world, with an age-standardised rate of 304/100,000 per year. The Caribbean region is reported to have the highest mortality rate of prostate cancer worldwide. Prostate cancer accounts for a large portion of the clinical practice for health-care practitioners in Jamaica. The Jamaica Urological Society is a professional body comprising 19 urologists in Jamaica who provide most of the care for men with prostate cancer in collaboration with medical oncologists, radiation oncologists, and a palliative care physician. The health-care system is structured in two tiers in Jamaica: public and private. The urologist-to-patient ratio is high, and this limits adequate urological care. Screening for prostate cancer is not a national policy in Jamaica. However, the Jamaica Urological Society and the Jamaica Cancer Society work synergistically to promote screening as well as to provide patient education for prostate cancer. Adequate treatment for localised prostate cancer is available in Jamaica in the forms of active surveillance, nerve-sparing radical retropubic prostatectomy, external beam radiation, and brachytherapy. However, there is a geographic maldistribution of centres that provide prostate cancer treatment, which leads to treatment delays. Also, there is difficulty in affording some treatment options in the private health-care sectors. Androgen deprivation therapy is available for treatment of locally advanced and metastatic prostate cancer and is subsidised through a programme called the National Health Fund. Second-line hormonal agents and chemotherapeutic agents are available but are costly to most of the population. The infrastructure for treatment of prostate cancer in Jamaica is good, but it requires additional technological advances as well as additional specialist services.

## Introduction

Jamaica is an island in the English-speaking Caribbean which boasts a population of 2.7 million inhabitants, with a male population of approximately 49%. The major ethnic group in Jamaica is African-Caribbean representing 91.2% of the population, and 6.2% reported being mixed. The gross domestic product (GDP) per capita in Jamaica is US$ 5471.72, and the island is classified as having a lower middle income economy.

In Jamaica, prostate cancer accounts for almost a third of all cancers diagnosed [1]. Arguably, Jamaica has the highest incidence of prostate cancer in the world, based on a reported rate of 304/100,000 per year by Glover in 1998 [2]. Consistent reports of the Jamaica Cancer Registry document that prostate cancer is the most common cancer overall as well as the most common cancer in men; however, the age-standardised incidence rate of 78.1/100,000 per year is much lower than that of Glover’s report [3]. It is also the most common cause of male cancer-related deaths, with an age-standardised mortality rate of 53.9/100,000 per year [4].

Treatment of prostate cancer is via multiple modalities and requires the collaboration of urologists, pathologists, medical oncologists, radiation oncologists, and palliative care physicians. This article summarises the state of prostate cancer in Jamaica as it relates to screening, treatment, and outcomes.

## Human resources and prostate cancer treatment

The Jamaica Urological Society was formed in 1993 and comprises 19 active members who are urologists trained either locally or overseas. Prostate cancer represents a large part of the clinical practice of locally practising urologists. It is therefore important to have adequate numbers of trained urologists to manage the large burden of men with prostate cancer.

Health care in Jamaica is two-tiered: public and private services. Urological public health care is available in Jamaica through four tertiary hospitals: University Hospital of the West Indies, Kingston Public Hospital, Cornwall Regional Hospital, and Mandeville Regional Hospital. The former two hospitals are located in the capital city, Kingston, in the south-eastern end of the island, and visiting patients represent about 22% of the total island public patient population. The University Hospital of the West Indies represents one of four campus sites of the regional university, the University of the West Indies, for undergraduate and postgraduate medical training. Seven urologists are affiliated with the University Hospital of the West Indies and Kingston Public Hospital combined. Cornwall Regional Hospital is located at the western end of the island, and four urologists are affiliated with this centre. Mandeville Regional Hospital is located in south-central Jamaica, and only one urologist is affiliated with the centre on a part-time basis. Private urological care is offered by all 19 urologists and is available in similar geographic regions as is public urological care in Jamaica. The ratio of urologist providers to males in the Jamaican population is approximately 1: 70,000. The skewed geographic distribution of urological services island wide as well as the low provider-to-patient ratio leads to a significant barrier in urological treatment in Jamaica.

The University of the West Indies (Kingston campus) has been offering postgraduate training in urology since 1996. There are currently 11 locally-trained practising graduates from this programme. Prior to this, locally practising urologists were trained either in the United Kingdom or Canada. The training programme is five years long, and there are presently seven urology residents in training. Residency training includes a one-year elective in a university-based urology training centre in the United Kingdom, Canada, or the United States of America. It is hoped that with continued training of residents, there will be a significant increase in urology provider status in order to treat the large volumes of patients with prostate cancer.

## Screening and prostate cancer in Jamaica

Though screening for prostate cancer is controversial internationally, high-risk groups such as black men are recommended to be screened annually for the disease [5]. This has been a recommendation of the Jamaica Urological Society since 1999. Men with a life-expectancy of 10–15 years are encouraged to have an annual digital rectal examination and blood taken to measure prostate specific antigen (PSA) levels.

PSA testing became available in Jamaica in 1989 [6]. Despite this, there is no formal national screening policy for prostate cancer in Jamaica. The most organised screening programme for prostate cancer in Jamaica is run through the Jamaica Cancer Society. The Jamaica Cancer Society is a non-profit, non-governmental organisation, which was formed in 1955, engaged in activities for the prevention and control of cancer in Jamaica. The society has a head office in Kingston with three additional satellite locations in north-central and south-central sections of Jamaica. The society commenced male screening clinics at its headquarters in 1995 with volunteer services by local urologists. Screening is provided at subsidised rates at multiple clinics weekly. In addition, the Jamaica Cancer Society has helped to build capacity in the public health-care system by donating an ultrasound machine to the University Hospital of the West Indies in 1999. This donation facilitated transrectal ultrasound-guided biopsies and was the first of its kind at the Urology Division in this hospital. The Jamaica Cancer Society and the Jamaica Urological Society have partnered together to increase prostate cancer awareness in Jamaica through regular health talks and fora, hosting of benefit concerts and plays, and regular contributions to the print and electronic media. Apart from the activities of the Jamaica Cancer Society, few patients present voluntarily for screening, however; most cases of prostate cancer are diagnosed through symptoms [7].

Barriers that delay or prevent African American men from being screened for prostate cancer include fear of the digital rectal examination, fear of a diagnosis of a medical disease, socioeconomic reasons, and fear of treatment-related adverse effects [8, 9]. These barriers may also be seen in the Jamaican male population and hinder screening. A cross-sectional study by McNaughton *et al*, examining attitudes toward prostate cancer screening in male medical doctors employed at the University Hospital of the West Indies, revealed that though most doctors were aware of the high prevalence of prostate cancer in Jamaica (97%) and agreed that screening should begin at 40 years (85%), only 44% of physicians encouraged screening for their patients and 41% of physicians have never had a personal digital rectal examination [10]. A similar study in rural Jamaica examining knowledge, attitudes, and practices towards prostate cancer screening in male health workers revealed that 71% of the health workers were aware of prostate cancer screening but only 27% had personally ever been screened [11]. Major barriers to screening in the sampled population were due to perceived discomfort of the digital rectal examination, male gender of the examining physician, and fear.

Screening has proven its efficacy in the United States population and resulted in a stage migration with 90% of cancers being diagnosed with clinically localised disease [12]. However in Jamaica, most cancers diagnosed are locally advanced or metastatic at presentation [7, 13, 14]. There is a great need therefore to have a national screening programme organised by the Ministry of Health in Jamaica.

## Treatment of prostate-localised prostate cancer in Jamaica

Recommended therapeutic options for localised prostate cancer of active surveillance, radical prostatectomy, interstitial prostate radiation (brachytherapy), and external beam radiation are available in Jamaica.

### Active surveillance

Active surveillance is practiced in Jamaica in eligible patients who have low-risk and low-volume prostate cancer. These are patients with Gleason score ≤ 6, PSA ≤ 10 ng/mL, and stage cT1c or cT2a, < 33% total positive cores at biopsy, and < 50% of single cores positive [15]. The aim of treatment is to monitor low-risk prostate cancer that may be clinically insignificant and avoid side-effects of active treatment such as urinary incontinence and erectile dysfunction. Treatment and follow-up protocols as described by Klotz *et al* are generally adhered to [16]. As expected in the Jamaican population of men with high-risk prostate cancer, many men are not currently on active surveillance treatment. In addition, we are wary of the extrapolation of results in studies with largely Caucasian populations to our high risk group. This fear was further strengthened by a recent report that suggested that African American men who had very low-risk prostate cancer and would have been eligible for active surveillance exhibited adverse pathologic features after radical prostatectomy [17]. A prospective trial investigating active surveillance in Jamaican men would be warranted to see the outcomes.

### Radical prostatectomy

In Jamaica, surgical treatment of localised prostate cancer is performed commonly via an open nerve-sparing radical retropubic approach as described by Patrick Walsh [18]. Laparoscopic radical prostatectomy was initially introduced in Jamaica in 2005 but is not performed as frequently as the open approach. Morrison *et al* reported on outcomes of radical prostatectomy performed at the University Hospital of the West Indies between 2000 and 2007 [19]. Oncological outcome was good, with a five-year biochemical-free survival rate of 78.4%. Due to deficiencies in chart reporting, there are no locally published reports on functional outcomes after radical prostatectomy (erectile function and urinary continence). However, major complications were rare in this series, and there was no perioperative mortality.

Ritch *et al* reported on an international comparative study of radical prostatectomy outcomes of Jamaican men with prostate cancer treated at the University Hospital of the West Indies and African American and Caucasian men treated at Columbia University Medical Centre [20]. The study revealed that Jamaican men presented at a slightly older age (61 years) for surgery than African American and Caucasian men. In addition, Jamaican men presented with higher PSA levels for surgery. Both Jamaican and African American men had worse pathological outcomes after radical prostatectomy compared to Caucasian counterparts. The five-year biochemical-free survival rate was similar for both African American and Jamaican men (76%, 74%) and was significantly less than in Caucasian men [20]. The findings suggested that possibly due to lack of organised screening locally, Jamaican men presented at a slightly older age with more advanced disease for surgical treatment of prostate cancer. However, oncologically, the outcome of Jamaican and African American men was worse than Caucasians; possibly due to genetic or molecular differences.

### Brachytherapy

Brachytherapy or interstitial prostate radiotherapy involves inserting radioactive seeds into the prostate under fluoroscopic and ultrasound guidance. This method of treatment is best for men who have low-risk prostate cancer with relatively small glands (<50 g). It may be given in combination with external beam radiation. Brachytherapy is only offered in the private health-care system in Jamaica at the Radiation Oncology Centre of Jamaica. It has been performed since 2004 under the mentorship of the late Dr Tom Shanahan. Seventy-five patients have been treated since its inception; however, there are no published data analysing outcomes. Due to its provision only in the private health-care system, there are challenges for the general uninsured population in being able to avail brachytherapy for treatment.

### External beam radiation

External beam radiation is available at two centres in the public health-care system (Kingston Public Hospital and Cornwall Regional Hospital). These centres provide machines which utilise cobalt technology for treatment of prostate cancer. The single machines at each centre also provide radiation for other cancers across the island. This leads to an untenable situation with long delays to commence treatment for prostate cancer, averaging 1–2 years. At the Radiation Oncology Centre of Jamaica, located in Kingston, a Varian Linear Accelerator machine has been used to administer external beam radiation in the private health-care system since 2001. Treatment at both facilities is with 78 Gy of radiation, with a maximum of 39 fractions for 5 days weekly for a total of 8 weeks.

The limited numbers of radiation facilities island wide as well as the poor geographic distribution of the existing centres presents an obstacle for availing treatment for patients. Intensity-modulated radiotherapy (IMRT) is not available in Jamaica. IMRT has largely replaced conventional conformal radiation therapy for prostate cancer worldwide due to its ability to reduce disease recurrence and morbidity compared to conformal therapy [21]. The absence of IMRT for prostate cancer treatment in Jamaica serves as a barrier to acceptable care.

## Treatment of advanced prostate cancer

Treatment of advanced prostate cancer requires a collaborative approach with urologists, medical oncologists, radiologists, neurosurgeons, and palliative care physicians. This usually requires the administration of androgen deprivation therapy, chemotherapy, potent analgesics, insertion of nephrostomies, or surgical or radiation treatment for skeletal-related events (bony metastases, spinal cord compression).

## Androgen deprivation therapy in Jamaica

Surgical castration via bilateral orchiectomy was the first method of androgen deprivation therapy (ADT) described for metastatic prostate cancer [22] and is performed in Jamaica. However, the National Health Fund (NHF), a government programme established to subsidise drug treatment in Jamaica has been effective in changing the pattern of drug usage for advanced prostate cancer. Prior to the introduction of ADT agents to the NHF in 2005, most men with locally advanced or metastatic prostate cancer were treated with surgical castration [13]. Conjugated oestrogens (Premarin®) were the commonest medications used at that time for androgen blockade. However, conjugated oestrogens are associated with thromboembolic side effects [23, 24] and surgical castration is associated with significant psychosocial and sexual side effects [25]. The commonest agents used currently for ADT in prostate cancer in Jamaica are the luteinising hormone, releasing hormone analogues (Goserelin) and anti-androgens (steroidal-cyproterone acetate and non-steroidal-bicalutamide) [13]. The NHF subsidy has enabled greater access to drugs which have improved health outcomes. Additional second-line agents (ADT and chemotherapy) available for castrate-resistant prostate cancer are ketoconazole, Taxol group of compounds, and abiraterone. However, the cost of these agents precludes widespread usage.

## Palliative care of prostate cancer in Jamaica

Palliative care is available formally through a single practising medical oncologist who provides ambulatory and limited in-hospital services. The Jamaica Cancer Society erected a 32-bed palliative care facility in Kingston in 1963 called the Hope Institute which provides inpatient care for persons with varied types of terminal cancers. The limited bed space of the Hope Institute and limited human resources providing palliative care for prostate cancer in Jamaica is a major shortcoming. The Jamaica Cancer Society provides additional assistance through its volunteer survivor programme which provides emotional support and disease information to patients with prostate cancer.

Collaboration occurs between urologists, medical oncologists, radiation oncologists, radiologists, and neurosurgeons in terminal prostate cancer care. Radiologists are well trained and equipped to provide upper urinary tract diversion via nephrostomy tubes in cases of distal ureteric obstruction by locally advanced or metastatic prostate cancer (metastatic pelvic lymph nodes).

Neurosurgeons commonly assist with patients seen with spinal cord compression or vertebral metastases by providing surgical decompression. Additionally, focal external beam radiation may be needed in these cases and is provided in the centres mentioned above.

## Conclusion

Prostate cancer is a major health-care burden in Jamaica. Screening for prostate cancer should be a national policy in Jamaica, coordinated by the Ministry of Health. This should ensure that all males over the age of 40 years have access to screening in both public and private facilities. Involvement of the family, particularly women, is imperative to promote prostate cancer screening and reduce the barriers to testing. This will be effective in downstaging prostate cancer at diagnosis, reducing mortality, and improving quality of life. Jamaica has good basic infrastructure for treatment of prostate cancer; however, there is need for increased numbers of urologists as well as improvement in technology for treatment. Continued collaborative research is needed in prostate cancer in Jamaica and translation of research findings into clinical practice and education of the community is imperative.

## Conflicts of interest

The authors have no conflicts of interest to declare.
